# Regenerative Therapies for Diabetic Microangiopathy

**DOI:** 10.1155/2012/916560

**Published:** 2012-03-29

**Authors:** Roberto Bassi, Alessio Trevisani, Sara Tezza, Moufida Ben Nasr, Francesca Gatti, Andrea Vergani, Antonio Farina, Paolo Fiorina

**Affiliations:** ^1^Nephrology Division, Transplantation Research Center (TRC), Children's Hospital, Brigham and Women's Hospital, Harvard Medical School, Boston, MA 02115, USA; ^2^DiSTeBA, Università del Salento, 73100 Lecce, Italy; ^3^Sorgente Research, 20127 Milan, Italy; ^4^Department of Biophysical and Medical Science, Higher Institute of Medical Technology, 1006 Tunis, Tunisia; ^5^Department of Medicine, San Raffaele Scientific Institute, 20132 Milan, Italy; ^6^Department of Obstetrics and Gynecology, University of Bologna, 40138 Bologna, Italy

## Abstract

Hyperglycaemia occurring in diabetes is responsible for accelerated arterial remodeling and atherosclerosis, affecting the macro- and the microcirculatory system. Vessel injury is mainly related to deregulation of glucose homeostasis and insulin/insulin-precursors production, generation of advanced glycation end-products, reduction in nitric oxide synthesis, and oxidative and reductive stress. It occurs both at extracellular level with increased calcium and matrix proteins deposition and at intracellular level, with abnormalities of intracellular pathways and increased cell death. Peripheral arterial disease, coronary heart disease, and ischemic stroke are the main causes of morbidity/mortality in diabetic patients representing a major clinical and economic issue. Pharmacological therapies, administration of growth factors, and stem cellular strategies are the most effective approaches and will be discussed in depth in this comprehensive review covering the regenerative therapies of diabetic microangiopathy.

## 1. Introduction

Diabetes represents one of the greatest medical and socioeconomic emergencies worldwide. Approximately 17.5 million people have been diagnosed with diabetes in the USA and their number is continuously growing by 1 million per year [1]. Hyperglycaemia, occurring in type 1 (T1D) and type 2 diabetes (T2D), is responsible for a wide number of complications, with the vascular ones representing the leading cause of morbidity and mortality in diabetic patients [2]. Accelerated arterial remodeling, atherosclerosis, and endothelial cell dysfunction, affecting the macro- and the microcirculatory system, are the main evidences and lead to progressive tissue hypoperfusion and hypoxia [3]. In diabetes, multiple actors concur in causing vascular remodeling, among them metabolic factors (e.g., hyperglycaemic and oxidative stress) which are important for chemical and biological modifications of the extracellular matrix, endothelial/vascular smooth muscle cells, and mechanical factors (e.g., wall shear and circumferential stress) due to the concomitant hypertension, which cause enhanced inward remodeling, paralleled by intima/media thickening, and attenuation of vessel dilation [4, 5]. Moreover, hyperglycaemia and advanced glycation end products (AGEs) have been shown to increase the matrix surrounding endothelial cells and vascular smooth muscle cells, increasing the deposition of proteins and the entrapment of molecules and reducing metalloproteinases activity, thus being responsible for impaired vessel dilation and wall stiffening [6, 7]. Interestingly, the UK Prospective Diabetes Study (UKPDS) and Diabetes Control of Complications Trial (DCCT) have found microvascular disease and hyperglycaemia intrinsically related [8]. Pharmacological therapies with anti-inflammatory and anti-AGE/ROS drugs, angiogenesis inhibitors, administration of growth factors, either as recombinant proteins or via gene transfer, and stem cellular strategies are the most effective approaches and will be discussed in depth in this comprehensive review covering the regenerative therapies of diabetic microangiopathy.

## 2. ***β***-Cell Replacement

Subcutaneous daily insulin injections improve glycometabolic control and HbA1c% levels; however they are not able to entirely halt the occurrence of diabetic complications [13]. *β*-cell replacement, either pancreas or islet transplantation, is a life-saving intervention which can restore normoglycaemia [19].

### 2.1. Pancreas Transplantation

Despite being a procedure still affected by major risks, pancreas transplantation, when successful, leads to an immediate insulin withdrawal, as the newly transplanted pancreas is capable to secrete insulin immediately after the revascularization, normalizing HbA1c% levels in the long term (up to 10 years) [20]. Diabetic retinopathy is the most frequent diabetic complication with at least 75% of patients with T1D developing the disease by 10 years and 40% of them degenerating into blindness within 3 years [21]. Studies performed in diabetic patients receiving pancreas or kidney-pancreas transplantation have shown that the normoglycaemia achieved with these approaches do not prevent or reverse diabetic retinopathy [9] (Table 1). Conversely, Wang et al. reported regression of diabetic nephropathy at 1 year in 43% of simultaneous kidney-pancreas-transplanted subjects [22]. In general, it has been shown that pancreas transplantation halts the progression of diabetic nephropathy [13, 23, 24] (Table 1). Studies performed on pancreas-transplanted patients have shown that diabetic glomerular lesions affecting their own kidneys prior to transplantation were not ameliorated by 5 years of normoglycaemia, while an improvement was observed after 10 years [12, 13] (Table 1). Different studies have shown that neuropathy progression (and the associated vascular degeneration) can be halted by successful pancreas transplantation [15, 25, 26]. Martinenghi et al. reported that progressive amelioration of nerve conduction velocity was prominently related to the pancreas graft, given that in a cohort of kidney-pancreas-transplanted patients, the failure of the pancreas, that occurred at least 2 years after successful combined transplantation, was associated with a deterioration of nerve conduction velocity back to pretransplant levels [27]. Coronary intimal thickness has been reported to regress in 40% of transplanted patients, as well as carotid atherosclerotic plaques have been seen to improve within 2 years after pancreas transplantation [28]. 

### 2.2. Islet Transplantation

Islet transplantation is a new concept *β*-cell replacement alternatively employed to pancreas transplantation in selected groups of patients suffering from severely poor glycaemic control and recurrent hypoglycaemic episodes, especially if associated with reduced hypoglycaemic awareness [8]. Lee et al. reported a stabilization of retinopathy in islet transplanted patients in some cases even at 1 year after transplant [10]. Venturini et al. investigated through color-Doppler-imaging the blood flow of the central retinal arteries in a group of islet-transplanted patients, before and at 1 year after transplantation, and found a significant increase in arterial and venous retinal blood flow velocities in transplanted patients compared to the control group [11] (Table 1). Islet transplantation is often performed in diabetic patients who have already experienced a kidney transplant due to end-stage renal disease; however, no unique interpretation about islet transplantation role on kidney graft function has been reached so far. Our group showed that in T1D patients islet transplantation is able to improve kidney graft survival and function, also decreasing microalbuminuria [14] (Table 1). Lee et al. studied the peripheral nerve function of islet transplanted patients demonstrating that *β*-cell replacement has a positive impact on polyneuropathy as well [10]. Accordingly, Del Carro et al. showed that islet transplantation may stabilize or even improve polyneuropathy by reducing AGE receptors (RAGEs) expression in formerly kidney-transplanted T1D patients [16] (Table 1). In the same work, skin biopsies from islet-transplanted patients showed a higher conservation degree of perineurium and vasa nervorum compared to end-stage renal disease and kidney-transplanted T1D subjects [16]. In a previous work, we similarly demonstrated that skin biopsies performed in successful islet transplanted patients, showed a stabilization of diabetic microangiopathy after 3 years of follow-up, with an increased expression of endothelial nitric oxide and von Willebrand factor, associated with a reduction of capillary basement membrane thickness and endothelial cellular swelling [17] (Table 1).

## 3. Pharmacological Therapies

### 3.1. Anti-Inflammatory Drugs

High glucose concentrations induce the production of the proinflammatory cytokine IL-1 in human pancreatic *β*-cells, contributing to impaired insulin secretion and *β*-cell proliferation [29]. In a randomized double-blind, trial, anakinra (a recombinant human IL-1 receptor antagonist) reduced HbA1c% and IL-6 concentration increasing C-peptide secretion in T2D patients [18]. Recent studies suggested that anti-inflammatory drugs may have an important role in diabetes therapy. The administration of salsalate (a prodrug carrying fewer side effects than aspirin) for a period of 2–4 weeks in T2D patients was able to reduce glucose blood concentrations while increasing insulin secretion [30–34]. Pentoxifylline is an anti-inflammatory methyl-xanthine derivative which is currently studied in a number of clinical trials evaluating its role in diabetic patients with nephropathy. It has been shown to significantly decrease proteinuria in both T1D and T2D patients; however given the small number of patients and the short duration of the studies, additional research is required to determine whether long-term use of pentoxifylline could be considered for the prevention or treatment of diabetic complications [35, 36]. Finally, it is becoming more and more evident that conventional therapies for diabetes are effective in reducing inflammation and improving diabetes outcomes via indirect or pleiotropic mechanisms. In fact, lifestyle modifications promoting weight loss, caloric restriction, and physical exercise, together with metformin and statin therapy, have recently been shown to reduce high C reactive protein levels in T2D patients [37, 38]. 

### 3.2. PKC Inhibitors

Hyperglycaemia is a fundamental metabolic factor involved in the development of both micro- and macrovascular complications, having numerous adverse effects such as the chronic activation of protein kinase C (PKC), a family of enzymes profoundly involved in the control of multiple cellular pathways [39]. Different PKC isoforms (PKC-*α*, -*β*1/2, and PKC-*δ*) have been shown to be associated with vascular alterations such as modifications of permeability, angiogenesis, synthesis of the extracellular matrix, cell growth/apoptosis, leukocytes migration, and cytokines production, thus leading to pathologies affecting the macrovasculature (atherosclerosis, cardiomyopathy) and the microvasculature (retinopathy, nephropathy, and neuropathy) [40]. Both preclinical and clinical studies using PKC-*β* inhibitors have been carried out, obtaining encouraging results. The PKC-*β* inhibitor ruboxistaurin (LY333531, RBX/Arxxant; Eli Lilly and Company, Indianapolis, IN) was employed in murine models of diabetes with virtually no effect on HbA1c%, blood glucose level, or blood pressure, however leading to normalization of glomerular filtration rate (GFR), urinary albumin, and TGF-*β*1 excretion and also reducing the glomerular and mesangial extracellular matrix [41, 42]. A multicenter pilot study evaluated the effect of LY333531 (32 mg/day) in T2D patients affected by diabetic nephropathy in addition to their current therapy with angiotensin-converting enzyme inhibitors and angiotensin receptor blockers, showing a 25% reduction in urinary albumin creatinine ratio (ACR) after 1 year and a conservation of the estimated GFR level [43]. The PKC-*β* inhibitor diabetic retinopathy study (PKC-DRS) reported a lower incidence of visual loss, need for laser treatment, and macular oedema progression in T1D and T2D patients treated with 32 mg/day LY333531 for 36–46 months [44, 45]. Inhibition of PKC-*β* through LY333531 is also beneficial to patients with symptomatic diabetic neuropathy, improving sensory symptoms and vibration sensation [46, 47]. A phase II multinational pilot study assessed the effect of LY333531 (32 or 64 mg/day for 1 year) in patients with diabetic neuropathy demonstrating a significant reduction of total symptoms and improvement at the vibration detection threshold [48]. Treatment with LY333531 (32 mg/day for 7 days) has been shown to prevent endothelium-dependent vasodilation abnormalities induced by hyperglycaemia as well [49]. 

### 3.3. AGEs and Oxidative Stress Strategies

Diabetes is characterized by abnormalities of mitochondrial ROS production that generate an increased oxidative stress in endothelial cells thus causing the development of diabetes complications [50]. In physiologic ageing AGEs can target proteins forming irreversible complexes that become resistant to proteolytic degradation; however, in the diabetic state the process results accelerated [51]. Aminoguanidine, scavenging intermediates in the glycation catalytic process, inhibits the formation of AGEs slowing the progression of diabetic nephropathy [52]; however the first clinical study performed in T1D patients with nephropathy and retinopathy, beside recording a consistent reduction of proteinuria, reported retinopathy worsening in some treated patients compared to placebo [53]. Vitamins of the B group represent another approach for the reduction of AGEs-related complications [54]. Preclinical studies revealed that pyridoxamine is effective in preserving kidney function in T1D and T2D murine models [54]. Consistently phase II clinical trials in proteinuric T1D and T2D patients showed a significant decrease in serum creatinine level, albumin, and TGF-*β* urinary excretion. An ongoing phase IIb study (http://clinicaltrials.gov/, NCT00734253) is evaluating the safety and efficacy of pyridoxine in nephropathic T2D patients. Finally, another clinical trial (http://clinicaltrials.gov/, NCT00565318) is now assessing the effect of benfotiamine, a lipophilic analogue of thiamine (B_1_ vitamin), on urinary albumin and *β*_2_-microglobulin excretion in T2D patients. Another class of therapeutic agents useful in reducing AGEs accumulation is advanced glycation cross-link breakers. Recently TRC4186 has been shown to decrease albuminuria and improve kidney function in diabetic mice with progressive cardiac and kidney failure, but 2-week treatment with ALT-711 (alagebrium), a novel AGE breaker compound had no effects on motor/nociceptive nerve dysfunction and vascular stiffness in diabetic mice after 8 weeks of diabetes [55, 56]. A clinical trial evaluating the effect of alagebrium (200 mg twice daily) on diabetic nephropathy (http://clinicaltrials.gov/, NCT00557518) reported a decrease in arterial pulse pressure and an increase in endothelial function and artery compliance [57, 58].

### 3.4. Inhibitors of Angiogenesis

Vascular endothelial growth factor (VEGF) is overproduced in the diabetic retina in response to capillary loss and/or microaneurysm formation; thus inhibition of   VEGF activity may play a pivotal role in the prevention of diabetes-related retinopathy [59]. Currently, there are three main anti-VEGF agents under investigation: pegaptanib sodium, a pegylated RNA aptamer that binds VEGF_165_ and the longer VEGF isoforms (Macugen; Eyetech Pharmaceuticals Inc, New York, and Pfizer Inc, NY); ranibizumab, a recombinant humanized monoclonal antibody specific for all human VEFG isoforms (Lucentis; Genentech Inc, South San Francisco, CA), and bevacizumab, another full-length humanized monoclonal antibody against all isoforms of human VEGF (Avastin; Genentech, South San Francisco, CA) [60]. VEGF has a role in the pathogenesis of diabetic nephropathy as well. SU5416 by selectively blocking all VEGF receptors at the level of the tyrosine kinase has been reported to ameliorate albuminuria in an experimental model of diabetic nephropathy [61]. In preclinical studies ruboxistaurin attenuated the effect of VEGF and so the progression of diabetic nephropathy [62]. 

## 4. Cellular Therapies

Stem cells have the unique ability to potentially originate any organ or tissue, being undifferentiated and capable of self-renewal [63]. Stem cells can be obtained from embryos, umbilical cord blood, and adult tissues (as bone marrow or adipose tissue) [63].

### 4.1. Cord Blood Stem Cells

Cord blood stem cells (CB-SCs) are a heterogeneous population composed of (i) very small embryonic-like stem cells, (ii) mesenchymal stem cells (MSCs), (iii) hematopoietic stem cells, and (iv) endothelial progenitor cells (EPCs) [63]. CB-SCs are easily collectable from 60–80 cc of umbilical cord blood and exhibit common features like the presence of long and highly preserved telomeres, the ability to form colonies when cultured *in vivo*, and a virtually absent oncogenic potential [63, 64]. An experimental study performed by Naruse et al. highlighted the therapeutic role for *ex vivo *expanded CB-EPCs in the treatment of diabetic neuropathy, showing that hind limb EPCs intramuscular injection into streptozotocin- (STZ-) induced diabetic rats improved muscles microvascular net, sciatic nerve conduction velocity, and endoneurial nutritive blood flow [65] (Table 2). Additionally, a clinical trial evaluating safety and efficacy of allogeneic CB-MSCs injections into pathologic lower limbs of T2D patients affected by peripheral arterial disease is currently ongoing at the Stem Cell Research Center at Qingdao University, (http://clinicaltrials.gov/, NCT01216865).

### 4.2. Mesenchymal Stem Cells

Among stem cells, great clinical interest is reserved to MSCs. Beside their potential to differentiate [66, 67], MSCs are characterized by strong hypoimmunogenic features, such as low expression of MHC-I-related antigens and absence of MHC-II antigens [68], making these cells immune privileged [69–77] (e.g., MSCs neither induce CD4^+^ activation nor are subjected to cell lysis induced by cytotoxic lymphocytes [78]). Moreover, MSCs are able to exert anti-inflammatory actions, for instance, by decreasing the secretion of TNF-*α* by dendritic cells [76, 77]. In a murine model of diabetic cardiomiopathy, Zhang et al. evaluated the ability of intravenous administration of BM-MSCs to either promote angiogenesis and mitigate ventricular remodeling, reporting an increased number of myocardial arterioles, an improvement of cardiac functionality, and a decrease of metalloproteinases activity, with a consequent smoothing of heart remodeling [79] (Table 2). Additionally, Wu et al. showed that allogeneic transplantation of BM-MSCs significantly promoted wound healing in diabetic *db/db* mice by accelerating reepithelialization and reconstituting capillary network density [80] (Table 2). Finally, Yang et al. showed that adipose-derived MSCs infusion in an STZ-induced murine model of diabetic retinopathy reduced blood glucose level and blood retinal barrier damage [81] (Table 2).

### 4.3. Endothelial Progenitor Cells

EPCs are circulating cells originating from several tissues such as bone marrow, peripheral blood, and cord blood, which are able to generate mature endothelial cells and vascular structures [82]. Thanks to their involvement in angiogenic and vasculogenic processes [82–86], EPCs represent an additional powerful tool for the treatment of diabetic microvascular complications. Interestingly, Barcelos et al. showed that human fetal aorta CD133^+^ progenitor cells, when transplanted into a murine model of diabetic wound, are either able to promote angiogenesis and to release VEGF-A in a high-frequency fashion [87]. Fiorina et al. demonstrated that the mobilization of endogenous BM-EPCs to the site of wound in a mouse model of diabetic wound healing is improved by the targeting of CXCR4/CXCL12 axis through a CXCR4 antagonist [88]. A currently ongoing phase I clinical trial is evaluating the efficacy and safety of the administration of AMD3100 (Plerixafor) and rhPDGF-BB (Becaplermin), two novel agents able to mobilize endogenous BM-EPCs, for the treatment of peripheral arterial disease in T2D patients, (http://clinicaltrials.gov/, NCT01353937). Recently, Asai et al. demonstrated that Sonic Hedgehog (SHh, a protein that stimulates BM-EPCs proliferation/migration and VEGF production, promoting the neovascularization of ischemic tissues) was responsible for wound healing acceleration in *db/db* mice by enhancing angiogenesis and recruiting BM-EPCs into the wound [89] (Table 2). Jeong et al. reported that hind limb BM-EPCs infusion was able to reverse diabetic neuropathy in STZ-induced diabetic mice, improving sciatic nerve conduction velocity and blood flow, compared to saline treated controls [90] (Table 2). Finally, transplanted BM-EPCs exhibited homing attitudes to the sciatic nerve and its vasa nervorum, also reporting a paracrine activity realized through the release of either angiogenic and neurotrophic factors [90] (Table 2). 

## 5. Gene Therapy

Growth factors administration by gene transfer is a promising approach for the treatment of diabetic microangiopathy, promoting endothelial cell proliferation, migration, and blood vessel formation.

### 5.1. Vascular Endothelial Growth Factor

VEGF is an endothelial-specific growth factor that promotes endothelial cells proliferation, differentiation, and survival, mediates endothelium-dependent vasodilation, induces microvascular hyperpermeability, and participates in interstitial matrix remodeling [91]. Notably, at low concentration VEGFs are mostly vasculoprotective, at high concentration have angiogenic effects, whereas at sustained high dose cause pathological angiogenesis [91]. VEGF-A, particularly its 165 isoform, plays a major role in vascular biology and is the first candidate for therapeutic applications *in vivo *[92, 93]. Isner et al. first demonstrated the tolerability of plasmid injection transferring a human VEGF_165_ encoding plasmid (ph) in patients with peripheral arterial disease [94, 95]. The safety and beneficial results of phVEGF_165_ were reported as well by Shyu et al. in patients with critical limb ischemia (CLI) [96]. Similarly, intramuscular phVEGF_165_ was tested in ischemic neuropathic patients [97]; however, no relevant biologic effects were obtained due to the partial inefficiency of VEGF gene transfer [98]. Conversely, a phase I study demonstrated the major efficiency of adenoviral (Ad) vector in VEGF_121_ transfer in severe vascular disease patients, showing an increase of lower-extremity flow reserve in response to acetylcholine [99]. Less optimistic outcomes were shown in other studies where Ad.VEGF_121_ administration in patients with intermittent claudication was not associated with an improvement in exercise performance and quality of life, and similarly, treatment with Ad.VEGF_165_ did not reduce the rate of amputation events [100] (Table 3). 

### 5.2. Fibroblast Growth Factors

Fibroblast growth factor (FGFs) is a large family of proteins capable of modulating the proliferation and migration of endothelial cells, fibroblasts, and smooth muscle cells [101]. FGF-1 is a potent mitogen for vascular cells and induces the formation of mature blood vessels *in vivo*. In 2007, Sanofi-Aventis started a promising trial (currently ongoing) based on the gene transfer of FGF-1 plasmid DNA in patients with CLI, known as TAMARIS (Therapeutic Angiogenesis for the Management of Arteriopathy in a Randomized International Study) [102], aiming at evaluating the efficacy and safety of intramuscular administration of NV1FGF in CLI patients, a plasmid-based gene delivery system for the local expression of FGF-1 [103, 104] (Table 3). After 1 year of follow-up, the primary endpoint of major amputations or death occurrence did not differ between the treated and the placebo arm [105]. FGF-4 has been shown to stimulate endothelial cells proliferation, migration, and neovascularization *in vivo* by upregulating endogenous VEGF-A expression [106]. Indeed, a currently ongoing clinical trial is evaluating the therapeutical potential of VEGF-A_165_/basicFGF delivery in the myocardium of refractory coronary artery disease patients [102]. So far, most efforts have concentrated on a single gene delivery therapy; however as multiple proteins are involved in the angiogenic process, efficiently delivering more than one gene might be a valuable approach [106].

### 5.3. Hepatocyte Growth Factor

Hepatocyte growth factor (HGF) is a large protein first identified as a potent hepatocyte mitogen and only lately discovered to stimulate endothelial cells growth [107]. Preclinical studies have shown that the delivery of HGF as a recombinant protein or naked plasmid induces therapeutic angiogenesis in rat or rabbit peripheral arterial disease models [108, 109]. Similarly, muscular injection of naked human HGF plasmid resulted in increased blood flow in the same models [109]. Following these preclinical results, the safety and efficacy of HGF plasmid administration was investigated in patients with CLI, evaluating limb tissue perfusion by transcutaneous oxygen tension measurement and reporting higher values of oxygen tension at 6 months as compared to controls [110] (Table 3).

### 5.4. Hypoxia-Inducible Factor-1

Hypoxia-inducible factor-1 (HIF-1) is a mesenchyme-derived pleiotropic transcriptional activator that acts as a regulator of oxygen homeostasis [111]. HIF-1 is a heterodimer composed of a constitutively expressed HIF-1*β* subunit and a O_2_-regulated HIF-1*α* subunit, with the latter increasing at low oxygen levels [111]. The complex of HIF-1*β*/*α* regulates the expression of many genes, including VEGF [111]. The injection of plasmid DNA encoding HIF-1*α* stimulates the recovery of blood flow in hind limb ischemia animal models [112, 113]. Similarly, intramuscular injection of adenovirus encoding functional HIF-1*α* in a murine model of hind limb ischemia increased the recovery of limb perfusion [113]. Finally, intramuscular injection of escalating dose of adenoviral HIF-1*α*-VP16 in patients with CLI has shown tolerability and safety [114] (Table 3).

### 5.5. Nerve Growth Factor

Recently, nerve growth factor (NGF) has been discovered to have cardiovascular protective roles and angiogenetic capabilities [115]. For these reasons, it is becoming particularly appealing for the treatment of diabetes vascular complications. Emanueli et al. strikingly introduced the concept of neurotrophins, particularly NGF, as autocrine proangiogenetic factors, promoting the growth of new capillaries and accelerating blood flow recovery in ischemic muscles of a mouse model of limb ischemia [116]. In fact, NGF seems to induce angiogenesis by increasing the expression level of VEGF-A and VEGF receptors [116, 117] and also by promoting NO production and metalloproteinases upregulation [116, 118]. Accordingly, Meloni et al. translated these findings into a gene therapy approach for the prevention of diabetic cardiomyopathy in a murine model of diabetes [119]. After 2 weeks of diabetes, mice were treated with NGF gene transfer via adenoassociated viral vectors [119]. Treated mice were found to be protected from myocardial microvascular rarefaction, hypoperfusion, increased deposition of interstitial fibrosis, and increased apoptosis of endothelial cells and cardiomyocytes [119].

### 5.6. Kallikreins

Kallikreins can be divided in tissue and plasma kallikreins differing in molecular weight, substrate specificities, and type of kinin released. Tissue kallikrein is a glycoprotein that stimulates cells to release autacoids such as nitric oxide and prostaglandins [120]. Circulating tissue kallikrein levels are increased in patients with peripheral obstructive vascular disease and in skeletal muscle after the induction of hind limb ischemia [121]. The angiogenic potential of tissue kallikrein in peripheral ischemia has been established in preclinical models using a gene-transfer approach [122]. Replication-defective adenoviral vector containing the human tissue kallikrein gene was injected into the adductor skeletal muscle of mice submitted to unilateral limb ischemia [123]. The successful transduction of the transgene resulted in ameliorated angiogenic response and haemodynamic recovery [123]. Emanueli et al. have also documented that human tissue kallikrein is able to prevent diabetic microangiopathy in STZ-induced diabetic murine models [124].

## 6. Conclusions

Multidisciplinary management of diabetes is of outstanding importance for the treatment and the prevention of diabetic complications, being helpful in reducing social costs and the detriment to the patient; however newer therapeutic approaches are needed. Novel mechanistic insights in the pathogenesis of endothelial dysfunction are rapidly being translated into new therapeutic opportunities; stem cells are expected to become promising therapeutic agents for diabetic patients due to their immunomodulatory characteristics, self-renewal, and differentiation ability; many clinical trials are demonstrating gene therapy as a valuable option to treat peripheral arterial disease. Nonetheless, gene therapy together with the cellular one is still waiting for randomized placebo-controlled double-blind, large-scale, clinical trials, ultimately defining their clinical role. Despite only short- and midterm follow-up data are available and long-term safety and efficacy end-points are required, some of these new strategies are close to become established therapeutic options, and some others hold in them the potential to halt diabetic complications.

## Figures and Tables

**Table 1:** Role of *β*-cell replacement in the treatment of diabetic microangiopathic complications divided by site of pathology. Islet and pancreas transplantation generally stabilize and in some cases improve the major diabetic complications in the long term. RAGE: advanced glycation end products receptor.

Complication	Pancreas transplantation	Islet transplantation	References
Ocular	Does not prevent diabetic retinopathy, while the reversal is still controversial	Stabilization of retinopathy, Increase in arterial and venous retinal blood flow velocity	Ramsay et al. N Engl J Med, 1988 [9]; Lee et al. Transplant Proc, 2005 [10]; Venturini et al. Transplantation 2006 [11]
Renal	Reduction in the thickness of the glomerular and tubular basement membranes, Decreased urinary albumin excretion	Retarded progression of diabetic nephropathy, Decreased urinary albumin excretion survival of the kidney graft	Fioretto et al. N Engl J Med, 1998 [12] and Lancet, 1993 [13]; Fiorina et al. J Am Soc Nephrol, 2003 [14]
Neurological	Progressive improvement of nerve conduction velocity	Positive impact on polyneuropathy, Reduce nerves' RAGE expression, Conservation of perineurium and vasa nervorum	Kennedy et al. N Engl J Med, 1990 [15]; Del Carro et al. Diabetes Care, 2007 [16]
Cardiovascular	Positive effects on atherosclerosis coronary and carotid intimal thickness reduction Peripheral vascular disease can worsen	Reduction in carotid intima media thickness Stabilization of microangiopathy in skin biopsies Improved dyastolic function Reduced hemostatic abnormalities	Fiorina et al. Diabetes Care, 2005 [17], Del Carro et al. Diabetes Care 2007 [16]; Larsen et al. Diabetes Care, 2007 [18]

**Table 2:** Overview of the experimental studies describing the role of stem cells in diabetic microangiopathy treatment and prevention. AD: adipose derived; BM: bone marrow; CB: cord blood; EPCs: endothelial progenitor cells; MSCs: mesenchymal stem cells; BRB: blood retinal barrier.

Complication	Cells	Outcomes	References
Neurological	CB-EPCs	Increased number of microvessels, Improved sciatic motor nerve conduction velocity, Increase of sciatic endoneurial nutritive blood flow	Naruse et al. Diabetes, 2005 [65]
Cardiovascular	BM-MSCs	Enhanced number of myocardial arterioles, Increase in fractional shortening, Mitigation of heart remodeling.	Zhang et al. Exp Clin Endocrinol Diabetes, 2008 [79]
Wound Healing	BM-MSCs	Acceleration of wounds healing, Increase in capillary density.	Wu et al. Stem cells, 2007 [80]
Ocular	AD-MSCs	Repair of BRB damages	Yang et al. Graefes Arch Clin Exp Ophthalmol, 2010 [81]
Wound Healing	BM-EPCs	Promotion of neovascularization.	Asai et al. Circulation, 2006 [89]
Neurological	BM-EPCs	Restoring of nerve conduction velocity, Increased blood flow in sciatic nerve, Increased nerves capillary density.	Jeong et al. Circulation, 2009 [90]

**Table 3:** Role of gene transfer in the treatment of diabetic microangiopathy. Gene transfer promotes endothelial cell proliferation, migration, and blood vessel formation. VEGF: vascular endothelial growth factor; FGF: fibroblast growth factor; HGF: hepatocyte growth factor; HIF-1*α*: hypoxia inducible factor-1*α*.

	Study	Year	Identifier	Site	Status/recruited pts
VEGF	Vegf gene transfer for critical limb ischemia	2010	NCT00056290	Steward St. Elizabeth's Medical Center of Boston	Completed
	VEGF gene transfer for diabetic neuropathy	2010	NCT00056290	Steward St. Elizabeth's Medical Center of Boston	Completed
	Angiogenesis using VEGF-A165/bFGF plasmid delivered percutaneously in no-option CAD patients; a controlled trial (VIF-CAD)	2009	NCT00620217	Institute of Cardiology, Warsaw, Poland	Completed
FGF	Efficacy and safety study of NV1FGF in patients with severe peripheral artery occlusive disease (TALISMAN202)	2010	NCT00798005	Sanofi-Aventis	Completed
	Efficacy and safety of XRP0038/NV1FGF in critical limb ischemia patients with skin lesions (TAMARIS)	2010	NCT00566657	Sanofi-Aventis	This study is ongoing, but not recruiting participants
HGF	Study of hepatocyte growth factor (HGF) via plasmid vector to improve perfusion in critical limb ischemia patients with peripheral ischemic ulcers	2011	NCT00189540	AnGes	This study has been completed
HIF	Safety and efficacy study of Ad2/Hypoxia inducible factor-1*α* (HIF-1*α*)/VP16 gene transfer in patients with intermittent claudication (WALK)	2010	NCT00117650	Genzyme	This study has been completed
